# A phase 1b study evaluating the effect of elacestrant treatment on estrogen receptor availability and estradiol binding to the estrogen receptor in metastatic breast cancer lesions using ^18^F-FES PET/CT imaging

**DOI:** 10.1186/s13058-020-01333-3

**Published:** 2020-09-11

**Authors:** Agnes Jager, Elisabeth G. E. de Vries, C. Willemien Menke-van der Houven van Oordt, Patrick Neven, Clasina M. Venema, Andor W. J. M. Glaudemans, Yamei Wang, Rebecca G. Bagley, Maureen G. Conlan, Philippe Aftimos

**Affiliations:** 1 Erasmus MC Cancer Institute, Post Office Box 2040, 3000 CA Rotterdam, Netherlands; 2 University Medical Center Groningen, University of Groningen, Hanzeplein 1, 9713 GZ Groningen, Netherlands; 3 Amsterdam UMC location VUMC, Cancer Center Amsterdam, De Boelelaan 1118, 1081 HV Amsterdam, Netherlands; 4 UZ KU-Leuven, Herestraat 49, 3000 Leuven, Belgium; 5 Radius Health, Inc., 950 Winter Street, Waltham, MA 02451 USA; 6 Clinical Trials Conduct Unit, Institut Jules Bordet – Université Libre de Bruxelles, Rue Héger-Bordet 1, 1000 Brussels, Belgium

**Keywords:** Advanced breast cancer, Elacestrant, Endocrine therapy, Estrogen receptor, 16α-^18^F-fluoro-17β-estradiol (FES), Hormonal therapy, Metastatic breast cancer, Positron emission tomography (PET), RAD1901

## Abstract

**Background:**

Elacestrant is an oral selective estrogen receptor (ER) degrader. This phase 1b open-label, non-randomized study (RAD1901-106) was initiated to determine the effect of elacestrant on the availability of ER in lesions from postmenopausal women with ER+ advanced breast cancer (ABC) using 16α-^18^F-fluoro-17β-estradiol positron emission tomography with low-dose computed tomography (FES-PET/CT).

**Methods:**

Eligible patients were postmenopausal women with ER+, HER2− ABC; tumor progression after ≥ 6 months of 1–3 lines of endocrine treatment for ABC; and measurable or evaluable disease. Two 8-patient cohorts were enrolled: one treated with 400 mg elacestrant once daily (QD) and one treated with 200 mg elacestrant QD with dose escalation to 400 mg QD after 14 days. Elacestrant was dosed continuously until progressive disease, toxicity, or withdrawal. FES-PET/CT was performed pre-dose at baseline and 4 h post-dose on day 14. The primary endpoint was the percentage difference in FES uptake in tumor lesions (maximum 20) after 14 days of treatment compared to baseline. Overall response was investigator-assessed by Response Evaluation Criteria in Solid Tumors [RECIST] version 1.1.

**Results:**

Patients (*n* = 16; median age, 53.5 years) had ABC with a median 2.5 prior lines of endocrine therapy. Median reduction in tumor FES uptake from baseline to day 14 was 89.1% (Q1, Q3: 75.1%, 94.1%) and was similar in both cohorts (89.1% [Q1, Q3: 67.4%, 94.2%], 200/400 mg and 88.7% [Q1, Q3: 79.5%, 94.1%], 400 mg). Residual ER availability (> 25% persistence in FES uptake) on day 14 was observed in 3 patients receiving 200/400 mg (3/78, 37.5%) and 1 patient receiving 400 mg (1/8, 12.5%). The overall response rate (ORR) was 11.1% (1 partial response), and clinical benefit rate (CBR) was 30.8%. Median percentage change in FES uptake did not correlate with ORR or CBR. Adverse events occurring in > 20% of the patients were nausea (68.8%), fatigue (50.0%), dyspepsia (43.8%), vomiting (37.5%), and decreased appetite, dysphagia, and hot flush (31.3% each). Most events were grade 2 in severity.

**Conclusion:**

Elacestrant 200 mg and 400 mg QD greatly reduced ER availability measured by FES-PET/CT. In a heavily pretreated population, elacestrant was associated with antitumor activity.

**Trial registration:**

ClinicalTrials.gov, NCT02650817. Registered on 08 January 2016

## Background

Hormone receptor-positive breast cancer accounts for approximately 70 to 80% of breast cancers [1, 2] for which endocrine treatment in both the adjuvant and advanced settings is recommended [3–5]. Available endocrine therapies decrease estrogen production (aromatase inhibitors), antagonize the estrogen receptor (ER) (selective estrogen receptor modulators [SERMs], e.g., tamoxifen), or degrade the ER (selective estrogen receptor degraders [SERDs], e.g., fulvestrant). Fulvestrant is the only approved SERD for the treatment of postmenopausal women with hormone receptor-positive advanced breast cancer. Fulvestrant has demonstrated valuable clinical benefit and prolonged progression-free survival (PFS) compared to anastrozole [6]. However, fulvestrant is limited by its pharmacokinetic (PK) properties and intramuscular route of administration, underscoring the need for novel ER antagonists that are efficacious and provide a more favorable PK profile [7, 8].

16α-^18^F-fluoro-17β-estradiol positron emission tomography (FES-PET) has been used in a variety of preclinical and clinical studies to detect ER expression in breast cancer. FES-PET is a non-invasive imaging modality that can be used to assess ER status of a tumor, potentially replacing tumor biopsy [9]. It can visualize ER occupation with SERMs and ER downregulation with SERDs due to reduced uptake of the FES tracer and has the potential to predict response based on the degree of downregulation [10–13]. A reduction of ≥ 75% was reported to be associated with a longer PFS in patients receiving fulvestrant compared to patients with a lower reduction in FES uptake (11.7 months vs 3.3 months, respectively; *P* < 0.05) [14].

Elacestrant is an investigational, nonsteroidal, oral SERD. In ER+ breast cancer cell lines, elacestrant showed dose-dependent ER degradation and inhibited estradiol-dependent induction of ER target gene transcription and cell proliferation [7, 8, 15]. In patient-derived xenograft models of heavily pretreated patients and in the ER+ MCF-7 breast cancer cell line xenograft model, elacestrant inhibited estradiol-activated tumor growth [7, 8, 15]. Elacestrant has also demonstrated antitumor activity in breast cancer models harboring mutations in estrogen receptor alpha gene (*ESR1*) known to confer resistance (e.g., Y537S, D538G) and those resistant to cyclin-dependent kinase 4,6 (CDK4/6) inhibitors [7, 16, 17].

In the clinical setting, elacestrant 400 mg daily has demonstrated an objective response rate (ORR) of 19.4% and median PFS of 4.5 months in a phase 1 trial (RAD1901-005) of heavily pretreated postmenopausal women with ER+/human epidermal growth factor receptor (HER)2− advanced/metastatic breast cancer (ABC/mBC) [18].

While the RAD1901-005 phase 1 trial was ongoing, the current phase 1b study (RAD1901-106) was initiated to determine the effect of elacestrant treatment on the availability of ER in lesions from patients with ABC using FES-PET with low-dose computed tomography (FES-PET/CT) imaging as a measure of ER downregulation. Additionally, the trial was designed to assess the safety, preliminary efficacy, correlation of ER availability with response, and PK of elacestrant in postmenopausal women with ER+ ABC.

## Methods

RAD1901-106 (NCT02650817) was a phase 1b, open-label, non-randomized, multicenter, international study conducted at 5 centers (3 in the Netherlands and 2 in Belgium) between February 2016 and August 2018. The study protocol and relevant supporting information were approved by the institutional review board at each participating site or by a national central review board. The trial was performed in accordance with ethical principles consistent with the Declaration of Helsinki and International Council of Harmonisation/Good Clinical Practice and applicable regulatory requirements. Each trial participant provided written informed consent.

The primary endpoint was the percentage difference in FES uptake in tumor lesions (up to a maximum of 20 lesions) after 14 days of treatment with elacestrant compared to baseline. Secondary endpoints were correlation of changes in FES uptake after elacestrant treatment to clinical responses measured by Response Evaluation Criteria in Solid Tumors (RECIST) v1.1 [19], preliminary antitumor effects of elacestrant, safety and tolerability of elacestrant, and elacestrant PK. An exploratory endpoint was correlation of tumor response with ESR1 mutations detected in circulating tumor DNA (ctDNA).

### Patients

Eligible patients were women ≥ 18 years of age who were postmenopausal (defined as > 56 years old with amenorrhea for > 12 months, < 56 years old with amenorrhea for > 12 months plus serum estradiol < 20 pg/mL and follicle-stimulating hormone > 40 mIU/mL, or prior bilateral ovariectomy). Patients had histologically proven ER+ (defined as ≥ 1% staining by immunohistochemistry [20]), HER2− ABC (either inoperable primary breast cancer or mBC); tumor progression after ≥ 6 months of 1–3 lines of systemic endocrine treatment for mBC; measurable disease according to RECIST v1.1 or evaluable disease; Eastern Cooperative Oncology Group (ECOG) performance status (PS) 0–2; life expectancy > 3 months; and adequate organ function. Prior CDK4/6 inhibitor therapy was allowed.

Exclusion criteria included liver-only metastases (due to high physiological liver uptake of FES, liver metastases are not evaluable by FES-PET/CT imaging); untreated or symptomatic central nervous system metastases; and prior treatment with tamoxifen or fulvestrant therapy < 42 days before first FES-PET/CT scan (since these agents may influence ER occupancy), other anticancer endocrine therapy < 14 days before first elacestrant dose, or chemotherapy < 28 days before first elacestrant dose. Full eligibility criteria are listed in an Additional file.

### Study procedures

The study consisted of a screening period of up to 21 days prior to the first dose of elacestrant (with the exception of tumor imaging tests, which were performed within 28 days of the first elacestrant dose); an open-label treatment period; and a follow-up period during which patients were followed for adverse events (AEs) and concomitant medication use for 30 days after the final dose of elacestrant or until resolution/stabilization of all AEs to grade ≤ 2.

A total of 8 patients were initially enrolled and treated with 400 mg elacestrant capsule once daily (QD)—the recommended phase 2 dose determined in study RAD1901-005. Following a protocol amendment, a second cohort of 8 patients was enrolled and treated with 200 mg elacestrant capsule QD for 14 days to assess target engagement of a lower dose that might be utilized when the 400-mg dose could not be tolerated alone or if elacestrant was given in combination with another agent. After 14 days, the dose was escalated to 400 mg QD. Elacestrant was dosed continuously with 28-day treatment cycles until progressive disease [PD], unacceptable toxicity, or patient/investigator decision to withdraw. Patients were instructed to take elacestrant at approximately the same time each day, approximately 30 min after a light meal; to fast for ≥ 1 h after dosing; and to remain upright for ≥ 2 h after taking elacestrant. After the first 14 days of treatment, dosing delays of ≤ 7 days were permitted. During the study, no hormonal medications, anticancer therapy, palliative radiotherapy, or strong CYP3A inducers or inhibitors were permitted.

### Assessments

At baseline, day 14 and day 28 of each cycle, and at end of treatment, patients underwent physical examination, ECOG PS assessment, and hematology and chemistry laboratory evaluations. Patients also underwent 12-lead electrocardiogram and coagulation evaluations at these time points, except day 14. Transvaginal ultrasound was performed at screening and end of treatment.

#### FES-PET/CT imaging

Imaging with FES-PET/CT was performed pre-dose at baseline and approximately 4 h post-dose (Tmax) on day 14 of cycle 1. Patients were given a single bolus injection of ~ 200 MBq FES prior to whole-body PET/CT imaging. Scan routines were conducted according to the European protocol for standardization of ^18^F whole-body PET studies, and quantification was performed using European Association of Nuclear Medicine (EANM) Research Ltd. (EARL) reconstruction according to the European standard for multicenter trials, as described in an Additional file [21, 22]. The percentage difference in background-corrected FES uptake in tumor lesions (up to an arbitrary maximum of 20 lesions) after 14 days of treatment with elacestrant compared to baseline was calculated. A relative decrease of < 75% in the median (background-corrected) tumor FES uptake and an absolute tumor lesion standardized uptake value (SUV)_max_ ≥ 1.5 was defined as incomplete reduction in ER availability.

#### ESR1 mutation analysis

Blood samples were collected for circulating tumor DNA (ctDNA) analysis at screening, day 28 of cycles 1–3, and at the end of treatment. The OncoBEAM™ assay (Sysmex Inostics, Baltimore, MD) was used to determine *ESR1* mutational status in ctDNA. This is a digital droplet PCR (ddPCR) assay that detects 12 mutations in the ligand binding domain of ESR1 (E380Q, S463P, V524E, P535H, L536H/P/Q/R, Y537C/N/S, D538G) [23].

#### Pharmacokinetic analysis

Blood samples were collected for PK analysis at baseline (pre-dose and 4 h post-dose), pre-dose on day 14 of cycle 1, and day 28 of cycles 1–3. Elacestrant plasma concentrations were determined using a validated ultra-performance liquid chromatography with tandem mass spectrometric detection method with a linear range of 0.05 to 100 ng/mL and a lower limit of quantification of 0.05 ng/mL (PRA Health Sciences, Assen, The Netherlands).

#### Tumor assessments

Tumor assessments were performed at screening, every 2 cycles, and end of treatment. Response was evaluated by the investigator using RECIST v1.1. Clinical benefit rates (CBR) at 16 weeks and 24 weeks were defined as the proportion of patients who had confirmed CR or PR any time during the study, or stable disease (SD) that lasted at least 16 and 24 weeks, respectively.

#### Safety assessments

Adverse events were collected throughout the study up to 30 days following the final elacestrant dose. The Medical Dictionary for Regulatory Activities version 17.1 was used for coding AEs, and the National Cancer Institute Common Terminology Criteria for Adverse Events version 4.03 was used to grade AE severity.

### Statistical methods

The study planned to enroll a total of 16 patients (8 per cohort). Percentage change in median FES uptake between baseline and day 14 was summarized as median and interquartile range due to slightly skewed data for each dose cohort and the overall population.

The intention-to-treat (ITT) population included all patients who received at least 1 full or partial dose of elacestrant. Best overall response was calculated for the response evaluable population, which included all patients who had measurable disease at baseline and at least 1 post-baseline RECIST assessment on any lesions (target or nontarget) and/or had a new lesion. Best overall response was summarized by dose cohort and reported as percentage with the 2-sided 95% exact confidence interval (CI) using the Clopper-Pearson method. The CBR was calculated for the clinical benefit evaluable population, which included all patients who had measurable and/or evaluable disease at baseline and at least 1 RECIST assessment post-baseline on any lesion (target or nontarget) and/or had a new lesion. Clinical benefit rate was summarized by dose cohort along with the 95% CI. Correlation between the median percentage change in FES uptake and best overall response was evaluated using the Spearman’s rank correlation coefficient. Kaplan-Meier methods were used to analyze PFS. Elacestrant plasma concentrations were summarized over time for each dose cohort. Adverse events and changes in vital signs, laboratory tests, and ECGs were summarized descriptively.

## Results

A total of 16 patients were enrolled and all patients received at least 1 dose of elacestrant and had paired baseline and day 14 FES-PET imaging. All patients have discontinued treatment, 12 (75.0%) due to radiographic or clinical disease progression, 3 (18.8%) due to an adverse event, and 1 (6.3%) for a protocol violation (treatment interruption > 7 days) (Additional file Fig. S1). Baseline characteristics were similar between the 2 dose cohorts (Table 1). The median age was 53.5 (range 43–84) years. All patients had ABC with a median number of prior lines of endocrine therapy of 2.5; no patients had prior CDK4/6 inhibitor therapy. Nine patients (56.3%) had *ESR1* mutation detected by ctDNA at baseline (D538G, *n* = 6; Y537S, *n* = 5; Y537C, *n* = 2; Y537N, *n* = 2; E380Q, *n* = 1; L536P, *n* = 1).

**Table 1:** Demographics and baseline characteristics

Characteristic	Elacestrant dose cohort		
	200/400 mg (***N*** = 8)	400 mg (***N*** = 8)	Overall (***N*** = 16)
Median age (range), years	57.0 (49, 74)	53.0 (43, 84)	53.5 (43, 84)
Female, *n* (%)	8 (100)	8 (100)	16 (100)
ECOG performance status, *n* (%)			
0	3 (37.5)	3 (37.5)	6 (37.5)
1	5 (62.5)	4 (50.0)	9 (56.3)
2	0	1 (12.5)	1 (6.3)
Ductal carcinoma, *n* (%)	8 (100)	8 (100)	16 (100)
Median time since breast cancer diagnosis (Q1, Q3), years^a^	12.5 (5.8, 16.7)	6.0 (4.2, 11.0)	8.5 (5.2, 13.7)
Stage IV, *n* (%)	8 (100)	8 (100)	16 (100)
Visceral disease^b^, *n* (%)	5 (62.5)	5 (62.5)	10 (62.5)
Bone-only disease, *n* (%)	1 (12.5)	1 (12.5)	2 (12.5)
*ESR1* mutation^c^, *n* (%)	4 (50.0)	5 (62.5)	9 (56.3)
Median number of lines of prior anticancer therapy (Q1, Q3), *n*			
Total, for ABC	3.0 (1.5, 3.0)	3.0 (2.0, 3.0)	3.0 (2.0, 3.0)
Endocrine therapy, for ABC	3.0 (1.5, 3.0)	2.0 (2.0, 3.0)	2.5 (2.0, 3.0)
Chemotherapy, for ABC	0 (0, 1.0)	0.5 (0, 1.0)	0 (0, 1.0)
Adjuvant endocrine therapy, *n* (%)	7 (87.5)	6 (75.0)	13 (81.3)
Prior fulvestrant, *n* (%)	4 (50.0)	2 (25.0)	6 (37.5)
Prior mTOR inhibitor, *n* (%)	3 (37.5)	3 (37.5)	6 (37.5)
Prior CDK4/6 inhibitor, *n* (%)	0	0	0

*CDK4/6*, cyclin-dependent kinase 4,6; *ECOG*, Eastern Cooperative Oncology Group; *mTOR*, mammalian target of rapamycin

^a^Median time since breast cancer diagnosis regardless of stage

^b^Includes the liver, lung, and pleura

^c^Mutations detected include D538G (*n* = 6), Y537S (*n* = 5), Y537C (*n* = 2), Y537N (*n* = 2), E380Q (*n* = 1), and L536P (*n* = 1)

### FES tumor uptake

**Fig. 1:**
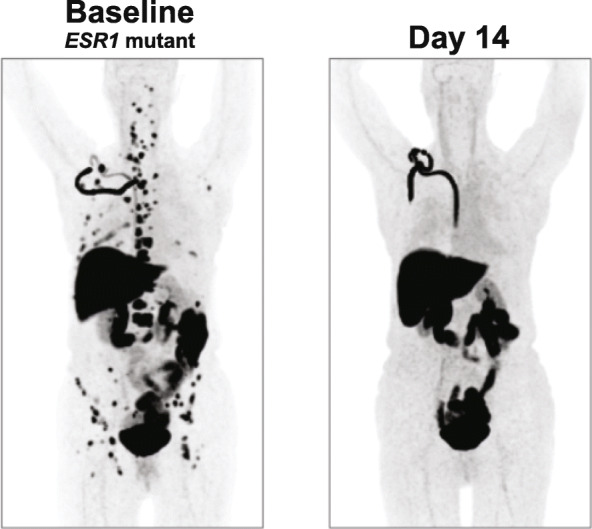
Reduction in FES uptake after elacestrant treatment in a patient with *ESR1* mutation. Images depict FES-PET scan in the patient at baseline (left) and 14 days after receiving elacestrant 400 mg daily (right), at which time a 96.6% reduction in FES uptake was observed. Physiologic FES uptake/excretion is observed in the liver, intestines, bladder, and port-a-cath infusion line; pathologic uptake is observed in bone lesions and lymph nodes

**Fig. 2:**
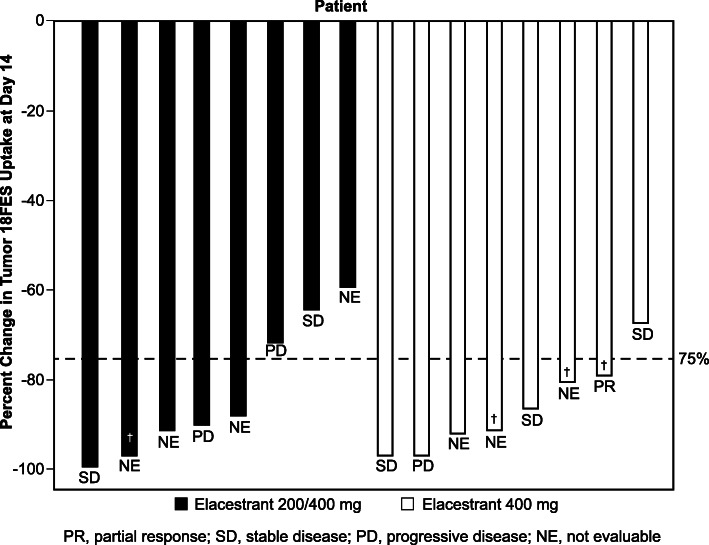
Percentage change from baseline to day 14 in median FES uptake for individual patients in the intention-to-treat population. One patient who had < − 100% change (i.e., > 100% reduction) was included as −100%. Response per RECIST (PR, SD, PD, NE) for each patient and patients with CBR at 24 weeks (†) are noted. FES, 16α-^18^F-fluoro-17β-estradiol

Percentage change from baseline in tumor FES uptake was assessed in all 16 patients. The median reduction in tumor FES uptake from baseline to day 14 was 89.1% (Q1, Q3: 75.1%, 94.1%; Table 2). Median reduction in FES uptake was similar in both dose cohorts (89.1% [Q1, Q3: 67.6%, 94.2%] in the 200/400 mg cohort and 88.7% [Q1, Q3: 79.5%, 94.1%] in the 400 mg cohort). The reduction in FES uptake was independent of baseline ctDNA *ESR1* mutation status (median reduction in FES uptake 90.2% [Q1, Q3: 80.2%, 96.4%] in patients with *ESR1* mutation and 86.3% [Q1, Q3: 71.3%, 91.2%] in patients without *ESR1* mutation). A representative pre- and post-elacestrant FES-PET scan in a patient with extensive metastases is shown in Fig. 1. Residual ER availability (defined as > 25% persistence in FES uptake) was observed in 1 patient in the 400-mg elacestrant cohort (1/8, 12.5%) and 3 patients in the 200/400-mg elacestrant cohort (3/8, 37.5%; Fig. 2). Among 9 patients with baseline *ESR1* mutation, 8 had at least 1 post-baseline *ESR1* result. Among these 8 patients, 5 had reduction in mutation allele frequency and 3 did not. There was no difference between percent change in FES uptake between the 2 groups (*P* > 0.05 calculated using the Wilcoxon rank sum test).

**Table 2:** Percentage change in FES uptake from baseline to day 14 for the two different dose cohorts

	Elacestrant dose cohort		
Parameter	200/400 mg (***N*** = 8)	400 mg (***N*** = 8)	Overall (***N*** = 16)
Number evaluated, *n* (%)	8 (100)^a^	8 (100)	16 (100)
Mean (SD), %	− 82.6 (15.5)	− 86.0 (10.2)	− 84.3 (12.8)
Median (Q1, Q3), %	− 89.1 (− 94.2, − 67.6)	− 88.7 (− 94.1, − 79.5)	− 89.1 (− 94.1, − 75.1)

*ITT* intention to treat, *FES* 16α-^18^F-fluoro-17β-estradiol

^a^One patient who had < − 100% change (i.e., > 100% reduction) was included as − 100%

### Antitumor efficacy

The ORR was 11.1% (Table 3); of 9 patients evaluable for overall response, 1 partial response was observed (in a patient without an *ESR1* mutation); the time to response was 7.9 weeks and the duration of response was 22 weeks. The CBR was 30.8%; 4 out of 13 patients evaluable for CBR had stable disease at 24 weeks. Of these 4 patients, 1 had an *ESR1* mutation. No significant correlation was found between the median percentage change in FES uptake and best overall response (Spearman’s rank correlation coefficient − 0.13; *P* > 0.7). The patient who experienced a partial response had a 78.9% reduction in FES uptake. In the ITT population, median PFS was 5.3 months (95% CI, 1.7, 17.9).

**Table 3:** Efficacy endpoints in evaluable populations

Parameter	Elacestrant dose cohort		
	200/400 mg	400 mg	Overall
Response	*N* = 4	*N* = 5	*N* = 9
ORR, %	0	20.0	11.1
Partial response, *n* (%)	0	1 (20.0)	1 (11.1)
Duration of response, weeks	–	22	22
Time to response, weeks	–	7.9	7.9
Stable disease, *n* (%)	2 (50.0)	3 (60.0)	5 (55.6)
Progressive disease, *n* (%)	2 (50.0)	1 (20.0)	3 (33.3)
CBR	*N* = 6	*N* = 7	*N* = 13
16 weeks, *n* (%)	3 (50.0)	4 (57.1)	7 (53.8)
24 weeks, *n* (%)	1 (16.7)	3 (42.9)	4 (30.8)
Median PFS (95% CI), months	3.6 (0.7, 17.9)	6.9 (0.7, NA)	5.3 (1.7 17.9)

*CBR* clinical benefit rate, *ORR* objective response rate, *PFS* progression-free survival

### Pharmacokinetics of elacestrant

**Fig. 3:**
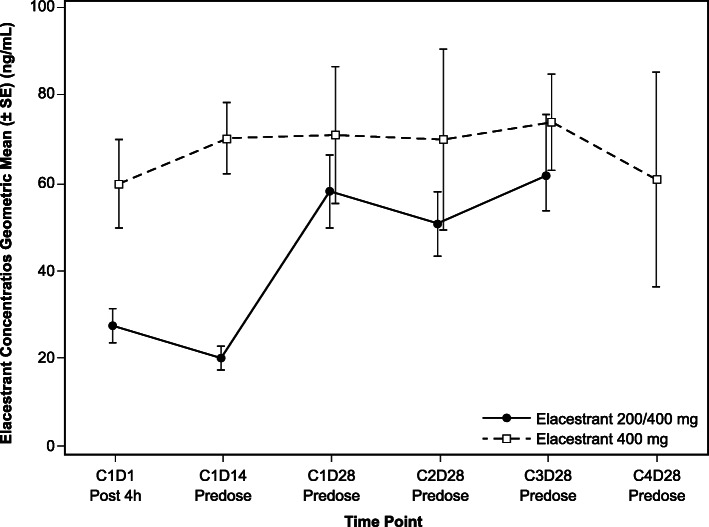
Geometric mean elacestrant plasma concentrations over time (*N* = 16). Vertical bars indicate standard error of the mean

Pharmacokinetics were evaluated in all 16 patients. Over the first 2 weeks of treatment, the geometric mean elacestrant plasma concentrations were approximately twice as high for patients receiving 400 mg QD as those for patients receiving 200 mg QD (59.8 ng/mL vs 27.4 ng/mL at cycle 1, day 1, hour 4 post-dose, respectively, and 70.1 ng/mL versus 20.1 ng/mL at cycle 1, day 14, pre-dose, respectively; Fig. 3). Elacestrant plasma concentrations at additional timepoints are summarized in Additional file Table S1.

### Safety

The median duration of elacestrant treatment was 4.3 months (range, 0.4–23.2). Patients in the 400-mg cohort remained on treatment longer than the 200/400-mg cohort (median 5.2 months [range, 0.7–23.2] vs 1.8 [range, 0.4–18.1] months, respectively). One patient receiving the initial dose of 200 mg did not dose escalate to 400 mg per protocol due to a serious AE of grade 3 esophagitis that was assessed as possibly related to elacestrant by the investigator and that resulted in treatment discontinuation. Two additional patients discontinued treatment due to AEs: grade 2 nausea, fatigue, and anorexia in 1 patient, and grade 2 cough and grade 1 upper extremity peripheral edema in 1 patient. Among all patients, 4 (25.0%) had dose delay and 2 (12.5%) had dose reduction due to a treatment-emergent AE (TEAE). All patients experienced at least 1 TEAE (Table 4). The most common TEAEs occurring in > 20% of patients were nausea (*n* = 11, 68.8%), fatigue (*n* = 8, 50.0%), dyspepsia (*n* = 7, 43.8%), vomiting (*n* = 6, 37.5%), decreased appetite, dysphagia, and hot flush (*n* = 5, 31.3% each; Table 4). The majority of patients (*n* = 10, 62.5%) had grade 2 TEAEs. Five grade 3 TEAEs occurred in 4 patients; no grade 4 or 5 events occurred (Table 5). Adverse events assessed as related to elacestrant by the investigator occurred in 15 patients (93.8%), with gastrointestinal disorders being the most commonly reported. No consistent trends were observed in clinically significant changes in laboratory parameters, vital signs, or electrocardiogram parameters.

**Table 4:** Adverse events occuring in ≥ 10% of the ITT population

Adverse event (AE), ***n*** (%)	Elacestrant dose cohort		
	200/400 mg (***N*** = 8)	400 mg (***N*** = 8)	Overall (***N*** = 16)
At least 1 AE	8 (100)	8 (100)	16 (100)
Nausea	5 (62.5)	6 (75.0)	11 (68.8)
Fatigue	4 (50.0)	4 (50.0)	8 (50.0)
Dyspepsia	2 (25.0)	5 (62.5)	7 (43.8)
Vomiting	5 (62.5)	1 (12.5)	6 (37.5)
Decreased appetite	4 (50.0)	1 (12.5)	5 (31.3)
Dysphagia	1 (12.5)	4 (50.0)	5 (31.3)
Hot flush	2 (25.0)	3 (37.5)	5 (31.3)
Hypertension	0	3 (37.5)	3 (18.8)
Arthralgia	1 (12.5)	2 (25.0)	3 (18.8)
Dizziness	2 (25.0)	1 (12.5)	3 (18.8)
Dyspnea	0	3 (37.5)	3 (18.8)
Abdominal pain upper	2 (25.0)	1 (12.5)	3 (18.8)
Back pain	0	2 (25.0)	2 (12.5)
Diarrhea	1 (12.5)	1 (12.5)	2 (12.5)
Esophageal pain	1 (12.5)	1 (12.5)	2 (12.5)
Neck pain	1 (12.5)	1 (12.5)	2 (12.5)
Pain in extremity	1 (12.5)	1 (12.5)	2 (12.5)
Cough	1 (12.5)	1 (12.5)	2 (12.5)
Nail discoloration	1 (12.5)	1 (12.5)	2 (12.5)
Anemia	0	2 (25.0)	2 (12.5)

*ITT* intention to treat

**Table 5:** Grade 3 adverse events, serious adverse events, and adverse events leading to discontinuation

Adverse event (AE), ***n*** (%)	Elacestrant dose cohort		
	200/400 mg (***N*** = 8)	400 mg (***N*** = 8)	Overall (***N*** = 16)
Grade 3 AEs^a^	1 (12.5)	3 (37.5)	4 (25.0)
Anemia	0	1 (12.5)	1 (6.3)
Circulatory collapse	0	1 (12.5)	1 (6.3)
Esophagitis	1 (12.5)	0	1 (6.3)
Cystitis	0	1 (12.5)	1 (6.3)
Pulmonary embolism	0	1 (12.5)	1 (6.3)
Serious AEs	1 (12.5)	2 (25.0)	3 (18.8)
Esophagitis	1 (12.5)	0	1 (6.3)
Amnesia	0	1 (12.5)^b^	1 (6.3)
Circulatory collapse	0	1 (12.5)^b^	1 (6.3)
Presyncope	0	1 (12.5)^b^	1 (6.3)
Pulmonary embolism	0	1 (12.5)^c^	1 (6.3)
Dyspnea	0	1 (12.5)^c^	1 (6.3)
AEs leading to elacestrant discontinuation	3 (37.5)	0	3 (18.8)
Esophagitis	1 (12.5)	0	1 (6.3)
Nausea	1 (12.5)^d,e^	0	1 (6.3)
Fatigue	1 (12.5)^d,e^	0	1 (6.3)
Anorexia	1 (12.5)^d,e^	0	1 (6.3)
Cough	1 (12.5)^d,f^	0	1 (6.3)
Upper extremity peripheral edema	1 (12.5)^d,f^	0	1 (6.3)

^a^There were no grade 4 AEs

^b^These events occurred in the same patient

^c^These events occurred in the same patient

^d^These events occurred during treatment with elacestrant 400 mg

^e^Grade 2 nausea, fatigue, and anorexia occurred in the same patient

^f^Grade 2 cough and grade 1 upper extremity peripheral edema occurred in the same patient

## Discussion

In the current study, the investigational oral SERD elacestrant decreased ER availability for binding 17β-estradiol to tumor metastases (assessed by reduction in FES uptake quantified using FES-PET/CT imaging) by a median of 89%, with 12 of 16 patients demonstrating a reduction of ≥ 75%. A reduction of ≥ 75% is suggested to be clinically relevant since this cut-off was found to be associated with a longer PFS in patients receiving fulvestrant compared to patients with a lower reduction in FES uptake [14].

In our phase 1 study, the proportion of patients with a ≥ 75% reduction in ER availability was 62.5% in the cohort receiving a dose of 200 mg elacestrant QD and 88% in the cohort receiving the recommended phase 2 dose (RP2D) of 400 mg QD. These proportions appear to be similar or higher than the 63% reported for treatment with fulvestrant [14], suggesting an equivalent or better reduction in ER availability by elacestrant at both the 200 mg and 400 mg doses. While the proportion of patients with a ≥ 75% reduction in ER availability with the elacestrant 200 mg dose at day 14 was lower than the 400 mg RP2D dose, this lower reduction in FES uptake should not lead to the conclusion that elacestrant 200 mg is not efficacious. It should also be noted that the reduction in FES uptake for 200 mg elacestrant was similar to what was reported for fulvestrant 500 mg at day 28 [14]; therefore, these data suggest that elacestrant 200 mg may be a potential option for patients who require a dose reduction from the 400 mg RP2D due to toxicity.

Patients enrolled in the current study were heavily pretreated (median 3 prior lines of therapy in all settings, median 2.5 lines of endocrine therapy in the advanced setting, 38% prior fulvestrant); however, none had prior CDK4/6 inhibitor exposure. Moreover, 56% of the patients had a detectable *ESR1* mutation by ctDNA at baseline, as is expected with extensive prior endocrine therapy [24, 25]. Despite these unfavorable prognostic characteristics, elacestrant demonstrated antitumor activity with an ORR of 11% and a median duration of response of 22 weeks. A 24-week CBR was observed in 31% of patients and the median PFS was 5.3 months. There was no correlation between reduction in ER availability to treatment response; however, the sample size evaluable for response in our study was too small to draw any conclusions (*n* = 9 evaluable for tumor overall response with 1 partial response). Other investigators have observed a correlation between degree of ER blockade on FES-PET and clinical response to tamoxifen and clinical benefit with fulvestrant [26, 27]. Dehdashti et al. reported a mean SUV decrease of 2.7 in responders versus 0.8 in nonresponders (*P* = 0.04) in a study of 11 women with newly diagnosed mBC [26]. Mortimer et al. reported a mean SUV decrease of 2.5 in responders versus 0.5 in nonresponders (*P* = 0.0003), which corresponded to a mean percentage decrease in SUV of 54.8% in responders versus 19.4% in nonresponders (*P* = 0.0003), in 40 women with locally advanced, chest-wall recurrent, or mBC [27]. It should be noted that neither study utilized RECIST criteria to define response or PET image reconstruction according to EANM/EARL guidelines. EARL/EANM accreditation is recommended for proper FES-PET implementation to avoid erroneous results [21, 22].

FES-PET scanning has not been routinely available at clinical sites and has generally been reserved for investigational purposes at select academic centers. As more data become available from ongoing trials validating the safety and predictive accuracy of FES-PET imaging, it could replace or supplement tumor biopsy. FES-PET allows the level and heterogeneity of ER expression across the full burden of disease to be evaluated to help guide treatment selection and characterize distinct subsets of patients [9]. The noninvasiveness of FES-PET could also facilitate serial imaging to monitor effect of ER-targeted therapies on treatment; ineffective agents could be switched earlier if reduction in ER availability is not observed. The first FES-PET imaging agent specifically indicated for use in patients with recurrent or metastatic breast cancer was approved by the FDA in May 2020 [28]. Availability of this agent in the US may lead to more widespread use for diagnostic purposes.

Strengths of this study are that ER target engagement was assessed with robust FES-PET/CT imaging, which allows whole-body visualization and quantification of ER availability on ER-expressing metastases and has shown predictive value for response on fulvestrant [12, 14]. The imaging was performed at an EARL/EANM-accredited academic center that routinely uses this technology. This study also assessed antitumor activity using the stringent RECIST criteria to measure response. The sample size was relatively modest for a phase 1 FES-PET/CT imaging study (only 8 patients were included per cohort), which limited the study’s capability to evaluate the correlation between reduction in ER availability and treatment response.

## Conclusions

This study demonstrated that elacestrant greatly reduces ER availability, as measured by FES-PET/CT imaging, at doses of 200 mg and 400 mg QD, to a similar, if not better, extent than that reported for fulvestrant. In a heavily pretreated population, including 56% of patients with *ESR1* mutation, elacestrant was associated with antitumor activity. The safety profile of elacestrant was acceptable, consisting predominately of low-grade upper gastrointestinal toxicity. The risk-benefit profile supports further development of elacestrant in ER+/HER2− ABC. This study supports the use of elacestrant 400 mg as the recommended dose for future studies. A randomized phase 3 study comparing elacestrant 400 mg to standard-of-care endocrine monotherapy (EMERALD: NCT03778931) in postmenopausal women and men with ER+/HER2− ABC is currently ongoing.

## Supplementary information

**Additional file 1: Table S1.** Elacestrant plasma concentrations over time. **Figure S1.** Patient disposition.

## Data Availability

Data that underlie the results reported in a published article may be requested for further research 6 months after FDA or EMA approval or 18 months after trial completion (whichever is latest). Radius will review requests individually to determine whether (i) the requests are legitimate and relevant and meet sound scientific research principles and (ii) are within the scope of the participants’ informed consent. Prior to making data available, requestors will be required to agree in writing to certain obligations, including without limitation, compliance with applicable privacy and other laws and regulations. Proposals should be directed to info@radiuspharm.com.

## Abbreviations

ABC: Advanced or metastatic breast cancer
AE: Adverse event
ctDNA: Circulating tumor DNA
CBR: Clinical benefit rate
CDK4/6: Cyclin-dependent kinase 4/6
CR: Complete response
CI: Confidence interval
CYP: Cytochrome P450
ECOG: Eastern Cooperative Oncology Group
ER: Estrogen receptor
*ESR1*: Estrogen receptor 1 (estrogen receptor gene alpha)
FES: 16α-^18^F-fluoro-17β-estradiol
HR: Hormone receptor
HER2: Human epidermal growth factor receptor 2
ITT: Intention to treat
mBC: Metastatic breast cancer
PK: Pharmacokinetic
ORR: Objective response rate
QD: Once daily
PR: Partial response
PET: Positron emission tomography
PFS: Progression-free survival
RECIST: Response Evaluation Criteria in Solid Tumors
SERD: Selective estrogen degraders
SD: Stable disease
SUV: Standardized uptake value
TEAE: Treatment-emergent adverse event
