# Supplementary material for: A phase 1b study evaluating the effect of elacestrant treatment on estrogen receptor availability and estradiol binding to the estrogen receptor in metastatic breast cancer lesions using 18F-FES PET/CT imaging
Source: Breast Cancer Res. 2020 Sep 11;22:97. doi: 10.1186/s13058-020-01333-3 (PMC7488419; doi:10.1186/s13058-020-01333-3)
Supplement: Supplementary file 1 — Additional file 1: Table S1. Elacestrant plasma concentrations over time. Figure S1. Patient disposition. [file 13058_2020_1333_MOESM1_ESM.pdf]

Additional File

A phase 1b study evaluating the effect of elacestrant treatment on estrogen receptor expression and estradiol binding to the estrogen receptor in metastatic breast cancer lesions using ^{18}F-FES PET

Agnes Jager, Elisabeth GE de Vries, C. Willemien Menke-van der Houven van Oordt, Patrick Neven, Clasina M. Venema, Andor W.J.M. Glaudemans, JungAh Jung, Rebecca G. Bagley, Maureen G. Conlan, Philippe Aftimos

Contents

Supplementary Methods**2**

Table S1. Elacestrant concentrations over time.**5**

Figure S1. Patient disposition.**6**

Supplementary Methods

Inclusion Criteria

For inclusion into the trial, patients were required to fulfill all of the following criteria:

1. Histologically-proven ER+, HER2- inoperable and/or ABC

   a. ER+ tumor was defined as ≥1% staining by immunohistochemistry (IHC) as defined in the 2010 American Society of Clinical Oncology (ASCO) recommendations for ER testing (Hammond et al, 2010)

   b. HER2- tumor defined as an IHC result of 0 or 1+ for cellular membrane protein expression, or an in situ hybridization negative result as defined in the 2013 ASCO recommendations for HER2 testing (Wolff et al, 2013)

2. Tumor progression after ≥ 6 months of at least 1 line of hormonal systemic treatment (selective ER modulator [SERM], selective ER degrader [SERD], or aromatase inhibitor) in the metastatic setting

3. Measurable disease according to RECIST criteria v1.1 (Eisenhauer et al, 2009); or evaluable disease. At least 1 non-irradiated lesion (measurable and/or nonmeasurable) must have been able to be accurately assessed by computed tomography (CT) or magnetic resonance imaging (MRI) or plain x-ray at baseline and follow-up visits

4. 18 years of age or older

5. Postmenopausal, defined as follows:

   - Greater than 56 years of age with amenorrhea for >12 months, or less than 56 years of age with amenorrhea for >12 months, serum E2 <20 pg/mL and follicle-stimulating hormone (FSH) >40 mIU/mL, or
   - Prior bilateral ovariectomy
   - Premenopausal patients who were medically induced to become postmenopausal for purposes of receiving endocrine therapy as standard of care treatment were eligible with documentation of appropriate lab testing

6. No prior treatment with elacestrant, GDC-0810, AZD9496, or other investigational SERD

7. Eastern Cooperative Oncology Group (ECOG) performance status 0-2

8. Life expectancy > 3 months

9. Resolution of all toxic effects of prior therapy or surgical procedures to Grade ≤ 1 (except alopecia)

10. Adequate organ function, defined as:

    a. Adequate bone marrow function

    - Absolute neutrophil count (ANC) ≥1.5 x 10^9/L
    - Platelet count ≥75 x 10^9/L

    b. Adequate hepatic function

    - Total bilirubin ≤1.5 x upper limit of normal (ULN) regardless of liver metastases. Inclusion of patients with increased serum indirect bilirubin (≤ 3 x ULN) due to Gilbert's syndrome was permitted

- Aspartate aminotransferase (AST), alanine aminotransferase (ALT) ≤3 x ULN, or AST and ALT ≤5 x ULN if liver metastases were present
- International Normalized Ratio (INR) <1.6 x ULN

c. Adequate renal function

- Creatinine ≤1.5 x ULN for institutional limits OR creatinine clearance > 60 mL/min/1.73 m^2 calculated by the Cockcroft-Gault formula for patients with creatinine above institutional normal

11. Written informed consent must have been given according to International Conference on Harmonization /Good Clinical Practice and national and local regulations

12. Able to comply with the protocol

Exclusion criteria

Patients meeting any of the following criteria were excluded from the study:

1. Pregnant or lactating

2. Severe concurrent disease, infection, co-morbid condition that, in the judgment of the investigator would have made the patient inappropriate for enrollment

3. Greater than 3 lines of endocrine therapy for metastatic disease

4. Prior anticancer treatment or investigational drug therapy within the following windows:

a. Tamoxifen or fulvestrant therapy <42 days before first FES PET scan

b. Any other anticancer endocrine therapy <14 days before first dose of study drug

c. Any chemotherapy <28 days before first dose of study drug

d. Any investigational drug therapy <28 days or 3 half-lives (whichever was longer) prior to first dose of study drug

5. Metastatic disease involving only the liver

6. Untreated or symptomatic central nervous system metastases. For patients with central nervous system metastases to be eligible, they must have completed radiotherapy at least 14 days prior to enrollment and required no steroid medication. If anticonvulsant medication was required, patients must have been stable on a non-enzyme-inducing anticonvulsant regimen

7. Known endometrial disorders, including evidence of endometrial hyperplasia, dysfunctional uterine bleeding, or cysts

8. Diagnosis of any secondary malignancy within 6 months prior to enrollment, except for adequately treated basal cell or squamous cell skin cancer, or carcinoma in situ

9. Any of the following within 6 months prior to enrollment: myocardial infarction, severe/unstable angina, ongoing cardiac dysrhythmias of Grade >2, uncontrolled atrial fibrillation of any grade, coronary or peripheral artery bypass graft, symptomatic cardiac failure, or cerebrovascular accident, including transient ischemic attack

10. History of any of the following blood disorders:

a. Coagulopathy, including history of deep vein thrombosis or pulmonary embolus within the past 6 months (except for adequately treated catheter-related venous thrombosis occurring > 1 month prior to the first dose of study drug)

b. Patients being treated with anticoagulant, eg, warfarin or heparin, were allowed to participate provided dose and coagulation parameters (as defined by local standard of care) were stable for at least 1 month prior to the first dose of study drug

11. Known human immunodeficiency virus infection, or active hepatitis C virus or hepatitis B virus infection

12. Impairment of gastrointestinal function or disease that may significantly alter the absorption of the study drugs (eg, ulcerative diseases, uncontrolled nausea, uncontrolled vomiting, chronic or uncontrolled diarrhea, malabsorption syndrome, gastric bypass, or small bowel resection)

13. Major surgery within 28 days prior to first dose of study drug

14. Local radiation therapy within 7 days prior to first dose of study drug

15. Use of strong CYP3A4/5 inhibitors and strong CYP3A4/5 inducers that could not be discontinued 7 days prior to the start and for the duration of study treatment

16. Endometrial thickness >11 mm

Supplemental FES-PET Methods

Low-dose CT for attenuation correction accompanied the FES-PET scan. Uptake of FES for was quantified using standardized uptake value (SUV) units, with FES uptake for individual lesions expressed as SUV_{max}. A threshold of SUV_{max} ≥1.5 was used to define FES-positive lesions at baseline. FES uptake was corrected for physiological background uptake using the unaffected contralateral side for symmetric structures, or adjacent tissue for asymmetric structures (normal organ SUV_{mean}). Liver lesions were excluded from analysis given the high physiologic background FES uptake in healthy liver tissue. When calculating the relative change in FES uptake, only FES-positive lesions were included.

Whole-body (head to mid-thigh) PET/CT was performed 60 min after tracer injection using a Siemens Biograph 40 or 64-slice mCT with a PET emission acquisition time of 3 min per bed position. Low dose CT was acquired for attenuation and scatter correction. Reconstructions of the scan and quantification were performed according to the European Association of Nuclear Medicine (EANM) guidelines for 18F imaging and the EANM Research Limited (EARL) criteria. All quantifications were performed on EARL reconstructed images with a 2 mm reconstructed spatial resolution. All scanners were EANM/EARL accredited at each site. Central review of imaging was done by Andor W.J.M. Glaudemans, MD, PhD.

References

Hammond MEH, Hayes DF, Dowsett M, et al. American Society of Clinical Oncology/College of American Pathologists guideline recommendations for immunohistochemical testing of estrogen and progesterone receptors in breast cancer. J Clin Oncol 2010; 28:2784-3543.

Wolff AC, Hammond MEH, Hicks DG, et al. Recommendations for human epidermal growth factor receptor 2 testing in breast cancer: American Society of Clinical Oncology/College of American Pathologists clinical practice guideline update. J Clin Oncol 2013; 31:3997-4014.

Table S1. Elacestrant plasma concentrations over time

Time Point	Elacestrant Dose Cohort	
	200/400 mg (n=8)	400 mg (n=8)
Cycle 1, day 1, hour 4 post-dose, n	7	8
Median (Q1, Q3), ng/mL	26.1 (18.8, 40.8)	64.1 (42.7, 85.5)
Geometric mean, ng/mL	27.4	59.8
Cycle 1, day 14, predose, n	7	8
Median (Q1, Q3), ng/mL	20.7 (15.5, 28.9)	80.9 (58.1, 87.1)
Geometric mean, ng/mL	20.1	70.1
Cycle 1, day 28, predose, n	6	7
Median (Q1, Q3), ng/mL	61.3 (44.0, 78.4)	75.5 (43.5, 101.0)
Geometric mean, ng/mL	58.1	70.9
Cycle 2, day 28, predose, n	4	6
Median (Q1, Q3), ng/mL	53.7 (39.9, 64.6)	68.0 (43.2, 77.1)
Geometric mean, ng/mL	50.6	69.9
Cycle 3, day 28, predose, n	3	6
Median (Q1, Q3), ng/mL	63.4 (48.5, 76.5)	77.1 (65.7, 82.3)
Geometric mean, ng/mL	61.7	73.8
Cycle 4, day 28, predose, n	-	3
Median (Q1, Q3), ng/mL	-	57.0 (31.4, 126.0)
Geometric mean, ng/mL	-	60.9
Cycle 5, day 28, predose, n	-	3
Median (Q1, Q3), ng/mL	-	63.3 (32.3, 107.0)
Geometric mean, ng/mL	-	60.3
Cycle 6, day 28, predose, n	-	1
Median (Q1, Q3), ng/mL	-	41.1 (41.4, 41.1)
Geometric mean, ng/mL	-	41.1

Figure S1. Patient disposition

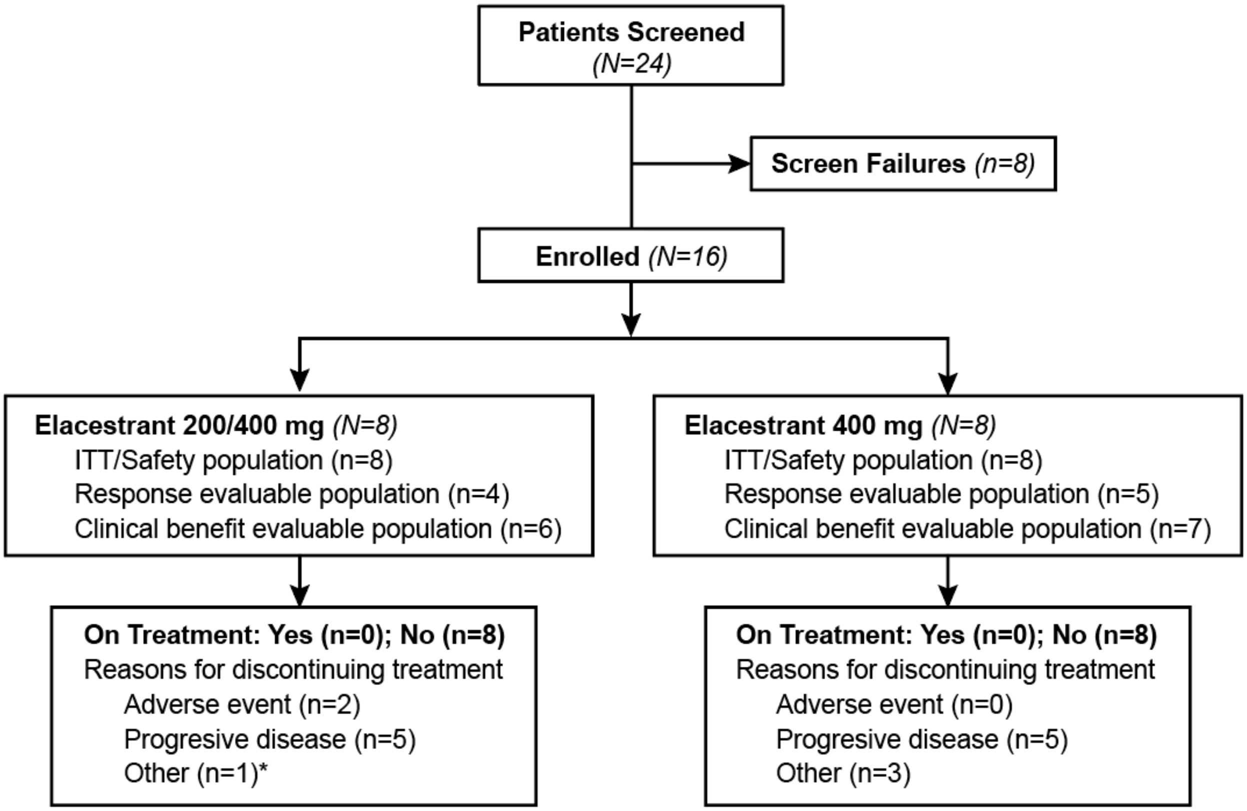

*One additional patient was discontinued from the study due to an AE, but was incorrectly recorded as "Other" category with discontinuation for progressive disease.

Other reasons include: clinical progression of disease; clinical progression; bone marrow involvement at bone marrow biopsy; and off study drug for more than the 7 days allowed per protocol.

ITT, intention-to-treat
